# Influence of Plug Rotational Speed on Microstructure and Texture of the Recrystallized Zone of a Friction Plug Weld Joint for AA6082-T6

**DOI:** 10.3390/ma15176011

**Published:** 2022-08-31

**Authors:** Defu Li, Xijing Wang

**Affiliations:** 1 State Key Laboratory of Advanced Processing and Recycling of Non-Ferrous Metals, Lanzhou University of Technology, Lanzhou 730050, China; 2 School of Materials Science and Engineering, Lanzhou University of Technology, Lanzhou 730050, China; 3 School of Railway Technology, Lanzhou Jiaotong University, Lanzhou 730070, China

**Keywords:** auxiliary heating, friction plug repair welding, preferential orientation, dynamic recrystallization

## Abstract

Experiments of friction plug weld AA6082-T6 aluminum alloy hole defects were carried out by using the method of friction auxiliary heating between the shaft shoulder and base metal. The grain refinement of the joint’s re-crystallized zone was significant, and there was obvious preferred orientation. Under the condition of other constant parameters, the rotational speed of the plug increases as the grain size of RZ increases and the component of High Angle Grain Boundaries decreases. The effect of a high deformation rate on dynamic re-crystallization is greater than that of high deformation temperature. The deformation texture component increased from 1600 r/min to 2000 r/min, while the re-crystallization texture component increased first and then decreased.

## 1. Introduction

Friction stir welding (FSW) is an environmentally friendly and efficient solid-phase welding technology, which is suitable for welding lightweight materials such as aluminum alloy. small residual stress after welding and high joint performance [1]. The friction stir welding will form a “keyhole” at the end of the weld, which will affect the overall performance of the joint [2]. Despite the fact that stirring needle pullback technology can fill the keyhole, it will make the bearing thickness of the joint thinner [3], affecting the overall joint performance. In industrial production, the “keyhole” is often eliminated by machining after adding a lead plate, while, for annular welding joints, “keyhole” is often inevitable [4].

Friction plug welding (FPW), a solid-state welding technology invented by the welding institute (TWI) of the UK in 1995, is considered as an ideal repair welding technique for keyhole type volume defects of Al alloys due to dominant advantages such as high joint strength, low residual stress and very small distortion over tungsten inert gas welding (TIG) methods [5]. Luan Guohong [6] analyzed the metallographies’ structure, hardness distribution and material flow mode of the LY12 aluminum alloy friction plug welded joint. The results show that during the process of friction plug welding, the material in the plasticized zone is recovered and re-crystallized, the grain is refined and the micro-hardness of the material is similar to that of the base material. Beamish [7] studied the friction plug welding process of 6082-T6 aluminum plate and obtained the influence law of welding parameters on welding quality. The ultimate tensile strength and fatigue strength of FPW joints for 2195 Al-Li are influenced by multiple factors such as a base metal strengthening mechanism, influence of friction stir welding process and plug materials [5,8]. High quality FPW welds could also be obtained on AA2219-T87 with optimized welding parameters, which mechanical properties were influenced by defects and micro-structural evolutions [9]. A sufficient volume of material from both the plug and a base metal flow upward and downward is critical to obtaining defect free weld [10]. Liu Jingxuan [11] prepared a 6005A-T5 aluminum alloy welded joint by the friction stir welding (FSW) process and studied the microstructure and mechanical properties of the joint. During the welding process, the β phase is completely dissolved into the aluminum matrix due to sufficient welding heat input in the weld core (NZ). The GP zone is formed during the subsequent natural aging process, leading to the hardness recovery of the NZ. Dynamic re-crystallization results in grain refinement, and the average grain size decreases with the increase in welding speed. A re-crystallized zone (RZ) with varied width is observed at the interface between the plug and base metal due to huge friction heat and deformation [12]. Softening is found near the bonding interface due to the disappearance of cold working and transformation of constituent particles. The tensile fracture morphology of the FPW joint is characterized by dimples.

The existing research results on the FSW head welding core zone have important reference significance for the research on the forming mechanism of the friction plug repair joint welding core zone. Zhang Liangliang [13] studied the grain morphology, grain boundary characteristics and texture composition evolution in the FSW zone of 6082-T6 aluminum alloy by the Electron Backscattered Diffraction (EBSD) technique. The results show that continuous dynamic re-crystallization occurs in the recertified side of the weld core zone, and the coarse grains of the base metal are refined to form shear texture. The rotational extrusion effect of the shaft shoulder makes the grain in the weld core zone rotate along the ND direction, forming the rotating cubic texture in the center of the weld core zone and forming the coexistence of shear texture and cubic texture on the forward side. Literature [14] has studied the influence of the spindle rotation speed of the microstructure evolution and mechanical properties of the friction stir welding joint in different areas. The grain boundary components have a small angle increase in the influence zone of the shaft shoulder, but have little change in the influence zone of the stirring needle and vortex zone. In the influence zone of the stirring needle and eddy current zone, grain refining and strengthening is the main mechanism, and the grain refining and strengthening effect decreases with the increase in rotating speed. The influence of ultrasound on dynamic re-crystallization of friction stir welding was studied in literature [15]. In the high-temperature welding core area, ultrasound promoted the nucleation rate and growth rate of equiaxed crystals, enhanced grain boundary dislocation movement and grain boundary self-diffusion, and promoted grain boundary sliding and migration, leading to the increase in growth rate of medium axial crystals in joint NZ.

**Figure 1:**
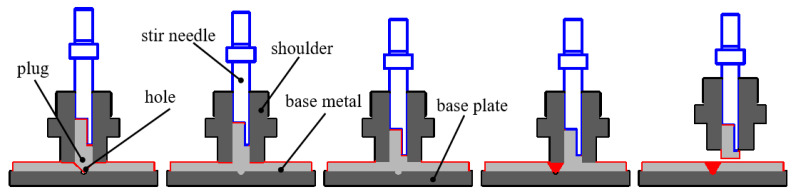
Schematic diagram of the FPW process by auxiliary heating of a shoulder.

Although the joints with good mechanical properties can be obtained under appropriate process parameters, the above FPW method has a high speed of plug (7500 r/min) and high top forging force (35–60 kN), so it also has high requirements for the FPW equipment [9,10,12]. In order to reduce the requirements on equipment, based on the filling friction stir welding [16] of the solid state connection principle, experiments of FPW for AA6082-T6 aluminum alloy were carried out by using the split welding tool and shoulder auxiliary heating [17]. The principle of auxiliary heating by the shoulder is shown in Figure 1. A concave lining hole is made on the base plate, and the plug can move axially relative to the shoulder. During the process of FPW, the heat of contact friction between the shoulder and the base metal was always maintained to provide a continuous auxiliary heat source. With the continuous feeding of the plug, some materials in contact with the plug and hole will produce plastic metal under the action of thermodynamic coupling. The plastic metal forms the initial FPW joint in the covering of the shoulder, the base plate and the cold base metal [18]. The feed of the plug is stopped until the plastic metal fills the entire hole of the friction stir weld joint, and then feeding occurs for another 3 s to form the top forging of the initial joint. After the top forging, the shoulder breaks the plug along the upper surface of base metal horizontally to form a smooth FPW joint.

**Figure 2:**
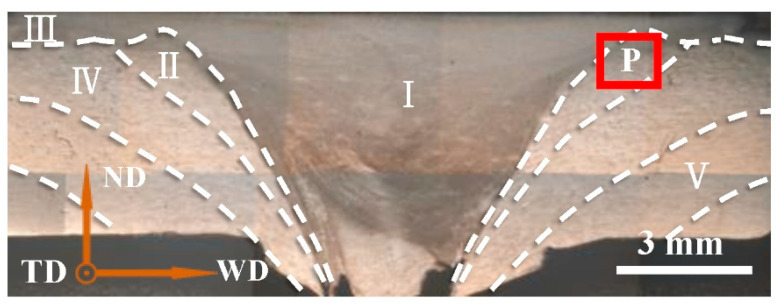
Schematic diagram of the cross-section morphology and partition and EBSD sample location (position P).

As is shown in Figure 2, one joint of FPW can be divided into five parts: nugget zone (NZ, I), re-crystallized zone (RZ, II), shoulder affected zone (SAZ, III), thermo-mechanically affected zone (TMAZ, IV) and the heat affected zone (HAZ, V). Among them, the RZ is the transition zone between the base metal and a plug, where the metal is plasticized under the action of thermodynamic coupling. The closer to the upper surface of the base metal, the less obvious the dividing line. Under the condition of another constant parameter, the tensile strength of the joint increases with the increase in the plug speed [19]. However, the mechanism from the influence in the plug speed on the forming of the joint is complex.

At present, the research on hole volume defects is mainly limited to the FPW process and joint performance [2,4,20,21,22,23,24,25]. However, the microstructure forming the mechanism of the FPW joint is complex. However, the friction plug repair welding of 6082 aluminum alloys by the shaft shoulder auxiliary heating method is different from friction stir welding, friction stir welding and friction plug repair welding technology and the process of the external energy field. On the other hand, because the plug rod is consumable material, the influence mechanism of thermal–mechanical coupling with thermoplastic during plug repair welding is different from that of friction stir welding. Therefore, the Electron Backscattered Diffraction (EBSD) technique was used to study the grain morphology, grain size and grain boundary characteristics of the FPW joints for AA6082-T6 aluminum alloy at a different plug speed [26]. It is of great significance to understand the forming mechanism of joint RZ.

## 2. Experimental Materials and Methods

AA6082-T6 aluminum alloy is used for the base metal and plug, and its composition is shown in Table 1. 9SiCr is used for the shoulder, and its composition is shown in Table 2. The size of the base metal is 150 mm × 100 mm × 5 mm, the diameter of the plug and hole is 10 mm. Based on previous experiments, the 80° equal angle was selected for the taper angle of the plug and hole, and the feed distance of the plug was 6 mm. Under the condition of other constant parameters, the plug rotational speeds of 1600 r/min, 1800 r/min and 2000 r/min were selected for the test, and well-formed plug repair joints were obtained. The cross-section morphology of the joint partition at 1800 r/min is shown in Figure 2.

The EBSD samples were processed at position P, which was 1 mm from the upper surface of joints. After the fine grinding, the samples were polished on the ion polishing instrument Leica EM TIC 3X. A field emission scanning electron microscope (Quanta450FEG) with EBSD probe (aztecx-max80) and a HKL Channel5 orientation analysis system were used for EBSD experiments. The sample tilt was 70° and the test voltage was 20 kV. The Low Angle Grain Boundary (LAGB) is shown by a white line, the black line represents the High Angle Grain Boundary (HAGB) and (hkl) [uvw] represents the texture.

## 3. Results and Analysis

### 3.1. Grain Morphology and Grain Boundary Characteristics of the Base Metal

**Figure 3:**
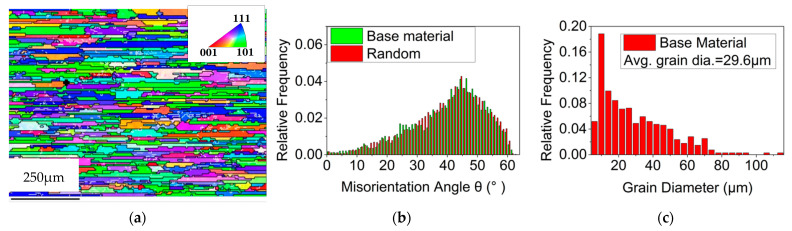
Grain morphology, misorientation angle and grain size of the base metal. (**a**) Grain morphology, (**b**) misorientation angle, (**c**) grain size.

As shown in Figure 3, the base metal has a typical rolling structure and has undergone complete re-crystallization after aging treatment. The misorientation angle is close to random orientation, and the grains have no obvious preferred orientation. The average grain size of the base metal is 29.6 µm.

### 3.2. Grain Morphology and Grain Boundary Characteristics of RZ

**Figure 4:**
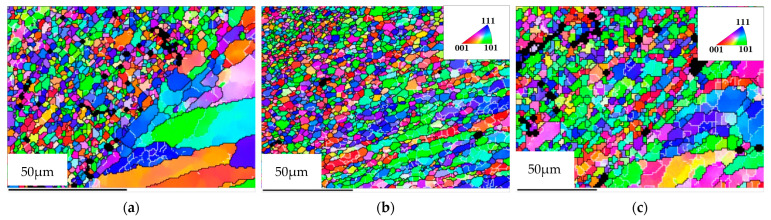
Grain morphology of RZ at different plug speeds. (**a**) 1600 r/min, (**b**) 1800 r/min, (**c**) 2000 r/min.

**Figure 5:**
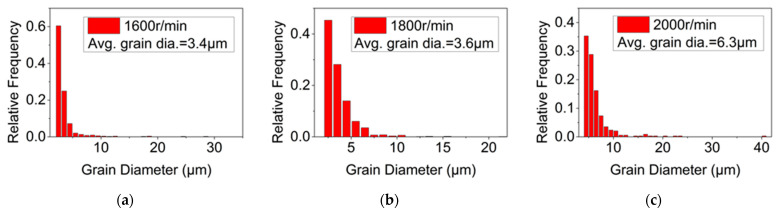
Grain size of RZ at different plug speeds. (**a**) 1600 r/min, (**b**) 1800 r/min, (**c**) 2000 r/min.

**Figure 6:**
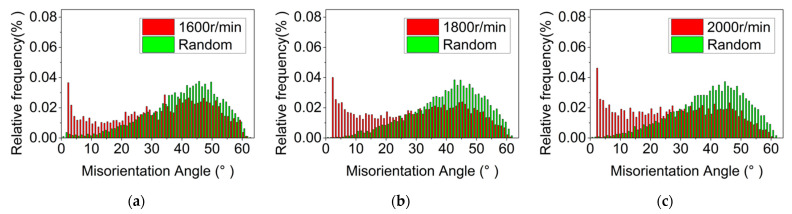
Misorientation angle of RZ at different plug speeds. (**a**) 1600 r/min, (**b**) 1800 r/min, (**c**) 2000 r/min.

Figure 4, Figure 5 and Figure 6 show the grain morphology, grain size and misorientation angle of RZ of FPW joints at different rotational speeds of the plug, respectively. Since RZ is the transition zone between the plug and base metal and from NZ to TMAZ, the shape of the grain changes from equiaxed to long strip. Therefore, the grain’s transitional characteristics are obvious in RZ. The average grain size of RZ increases with the increase in rotational speed of a plug when other parameters are constant. The misorientation angles of the RZ deviate from random orientation distribution, and there are no obvious preferred orientations.

As the plug is being fed, the material in contact with the plug and hole is plasticized, which increases dislocation and forms sub-grains [27]. Under the action of thermal activation energy, sub-grains form large grains by rotation, and continuous dynamic re-crystallization occurs [28]. Under the condition of other constant parameters, the higher the speed of the plug, the higher the temperature of the friction plug welding process. The higher the deformation temperature, the easier the grain boundary diffusion and migration, thus promoting the grain growth. Therefore, the rotational speed of the plug increases from 1600 r/min and 1800 r/min to 2000 r/min and, accordingly, the average grain size of the RZ increases from 3.4 µm and 3.6 µm to 6.3 µm.

**Figure 7:**
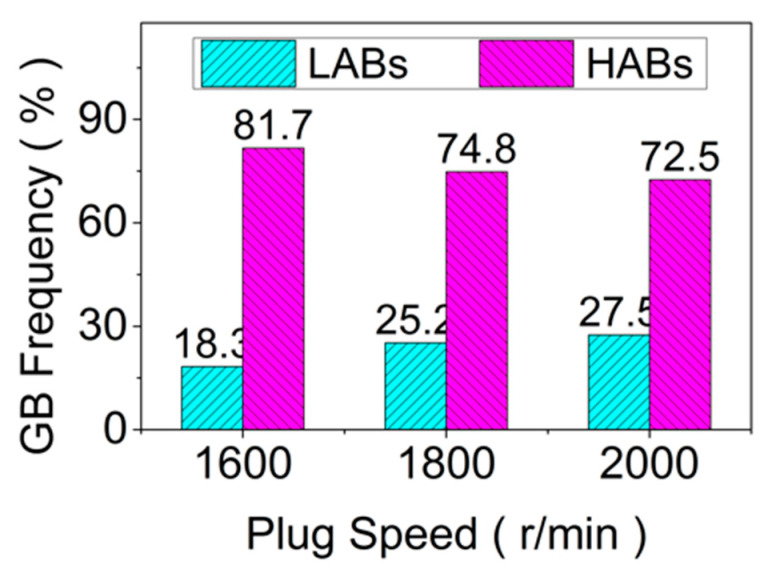
Components of LAGB and HAGB at different plug speeds.

In general, when the deformation temperature is higher, the thermal vibration and diffusion of atoms in the alloy increase and the nucleation rate of dynamic re-crystallization increases, which is conducive to the transformation from LAGB into HAGB [29]. The RZ is at the maximum torque of plug rotation, and the rotation of the plug will cause the breakage of the re-crystallized grains. The higher the rotational speed, the higher the deformation rate, and the more obvious the crushing effect. Therefore, compared to the high deformation temperature, the high strain rate in RZ has a greater influence on dynamic recrystallization [30]. The higher the rotational speed, the greater the crushing effect and the more unfavorable for the nucleation of new grains [31]. The higher the strain rate, the more dislocation, which is generated during the deformation process, and the higher the LAGB component, as shown in Figure 7.

**Figure 8:**
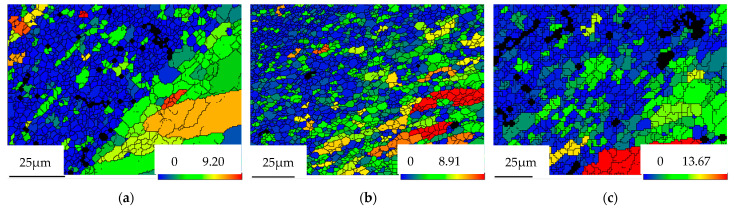
GOS diagram of RZ at different plug speeds. (**a**) 1600 r/min, (**b**) 1800 r/min, (**c**) 2000 r/min.

As is shown in Figure 8, it can be seen from the GOS (Grain Orientation Spreads) diagram of RZ at different plug speeds that the dislocation density of RZ increases with the increase in the plug rotational speed [32]. This indicates that under the condition of other certain process parameters, the increase in the plug rotational speed will lead to the rise in the temperature during the FPW process, and it is beneficial to the dynamic recrystallization [33]. However, since the RZ is located at the joint surface of the plug and hole, the deformation rate changes the most during the plug’s repaired welding. Therefore, the effect of increasing the deformation rate for RZ is greater than that of increasing temperature. The higher the rotational speeds of the plug and the higher the component of LAGB, the higher the density of dislocation. This is consistent with the above analysis.

### 3.3. Texture Types and Components of RZ

In order to further study the grain orientation evolution of the joint interface, HKL Channel 5 was used to calculate the preferred grain orientation of the RZ at different rotational speeds.

**Figure 9:**
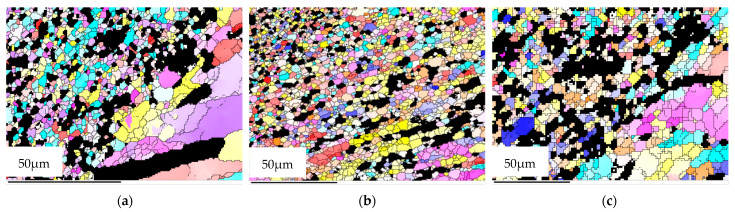
Grain chromatic aberration and distribution of different textures. (**a**) 1600 r/min, (**b**) 1800 r/min, (**c**) 2000 r/min.

Table 3 shows the texture and components of the RZ at different rotational speeds. At 1600 r/min, the re-crystallized Cube, (112) [$\bar{1}\bar{1}$1] and (011) [100] deformation textures are mainly present in RZ, while at 1800 r/min, the textures are mainly re-crystallized Cube, P and (313) [$\bar{1}$01] deformation textures. At 2000 r/min, the textures are mainly (313) [$\bar{1}$01] deformation texture, Goss texture and Copper texture [34]. Grain chromatic aberration and distribution, corresponding to different textures, are shown in Figure 9.

The rotational speed of the plug has an important effect on the texture and distribution of the RZ. In the process of FPW by shaft shoulder assisted heating, the higher the speed of the plug, the higher the temperature of the shoulder assisted heating and the higher the grain deformation rate of RZ. Moreover, the effect of a high deformation rate for dynamic recrystallization is greater than that of the high deformation temperature. Dynamic recrystallization is a rate-controlled process. The deformation rate not only affects the grain size and nucleation rates of new grains, but also causes the nucleation breakage of new grains of a high deformation rate. The higher the rotational speed of the plug and the higher the deformation rate, the more obvious the crushing effect. Therefore, with the increase in speed from 1600 r/min to 2000 r/min, the deformation texture component of RZ increases, while the recrystallization texture component increases first and then decreases.

## 4. Conclusions

The friction plug welding of AA6082-T6 aluminum alloy was carried out by the shaft shoulder’s auxiliary heating. Under the conditions of matching the 80° cone angle of the plug and a hole and a 6 mm feed rate, well-formed FPW joints were obtained. The grain morphology, grain boundary characteristics and texture components of the joints RZ at 1600 r/min, 1800 r/min and 2000 r/min were studied by the electron backscatter diffraction technique (EBSD). The following conclusions were drawn from this study.

(1) In RZ, there are the transitional characteristics and the grain refinement. When the other parameters are constant, the grain size increases with the increase in rotational speeds. The misorientation angles deviate from random orientation, and there is an obvious preferred orientation.

(2) The average grain size increases with the increase in the rotational speed from 1600 r/min to 2000 r/min.

(3) With the increase in rotational speed from 1600 r/min to 2000 r/min, the deformation texture component increases, while the re-crystallization texture component increases first and then decreases.

## Figures and Tables

**Table 1:** AA6082-T6 aluminum alloy material composition (mass fraction, %).

Si	Fe	Cu	Mn	Mg	Cr	Zn	Ti	AL
0.89	0.3	0.04	0.58	0.93	0.06	0.04	0.01	BAL

**Table 2:** 9SiCr composition (mass fraction, %).

C	Si	Mn	S	P	Cr	Ni	Cu	Fe
0.85–0.95	1.2–1.6	0.30–0.60	≤0.03	≤0.03	0.95–1.25	≤0.25	≤0.3	BAL

**Table 3:** Texture types and components of RZ at different rotational speeds.

Color Code	(hkl) [uvw]	1600 r/min	1800 r/min	2000 r/min
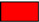	(001) [100]	12.9	15.5	5.95
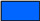	(214) [$\bar{1}\bar{2}$1]		9	9.47
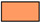	(011) [1$\bar{2}$2]		13.5	
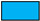	(011) [100]	15.7	8.19	12.8
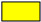	(313) [$\bar{1}$01]	15.4	24.2	20.6
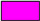	(111) [$\bar{1}$01]	10.5	11.4	12.5
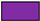	(112) [$\bar{1}\bar{1}$1]	18.7		12.5
